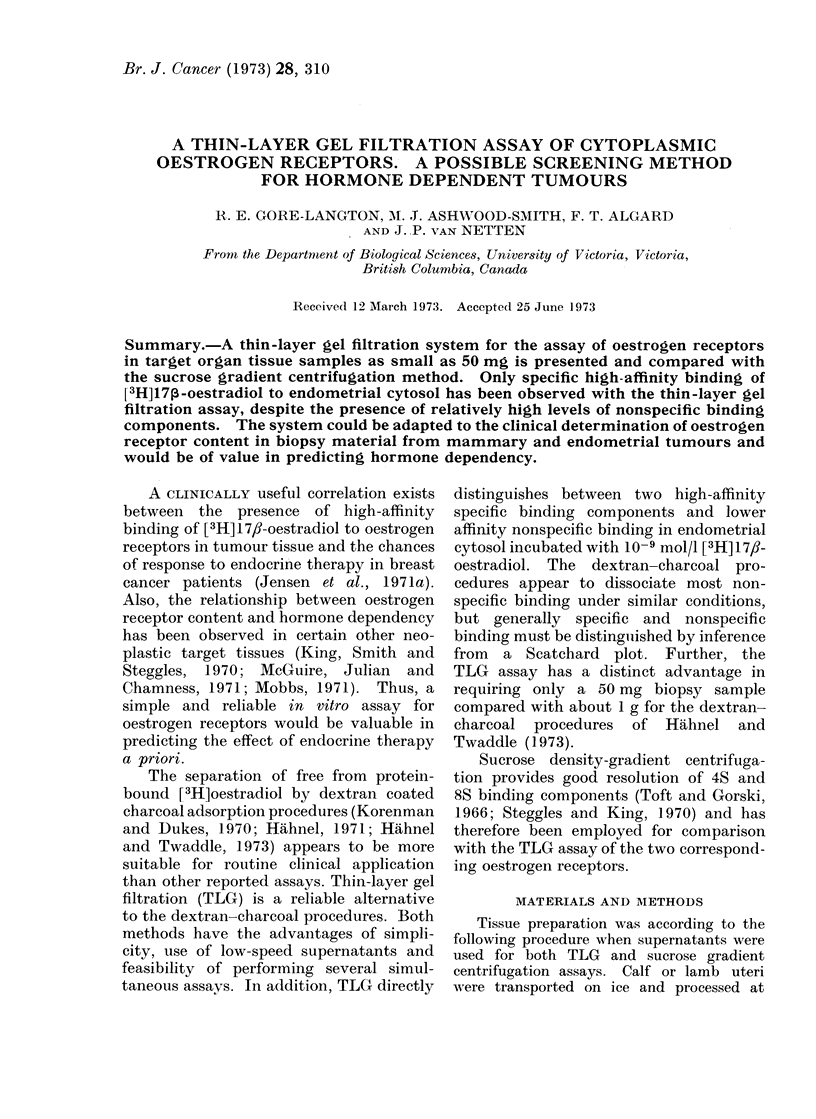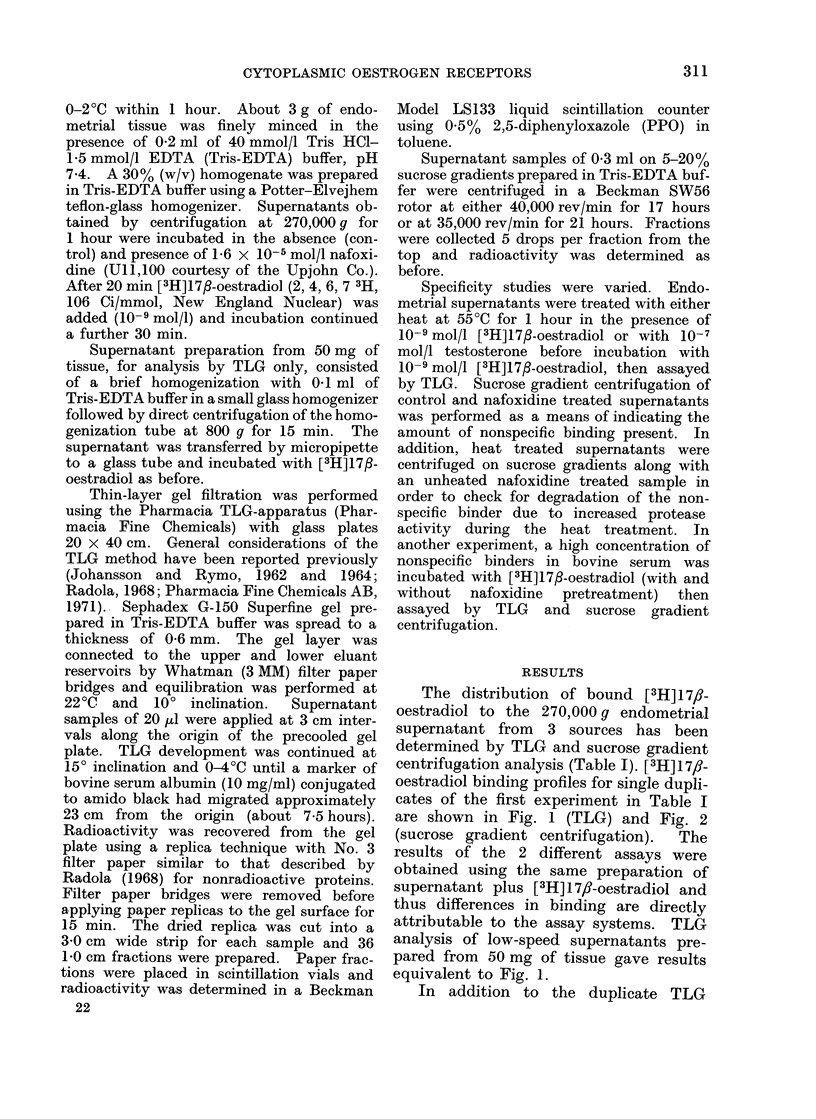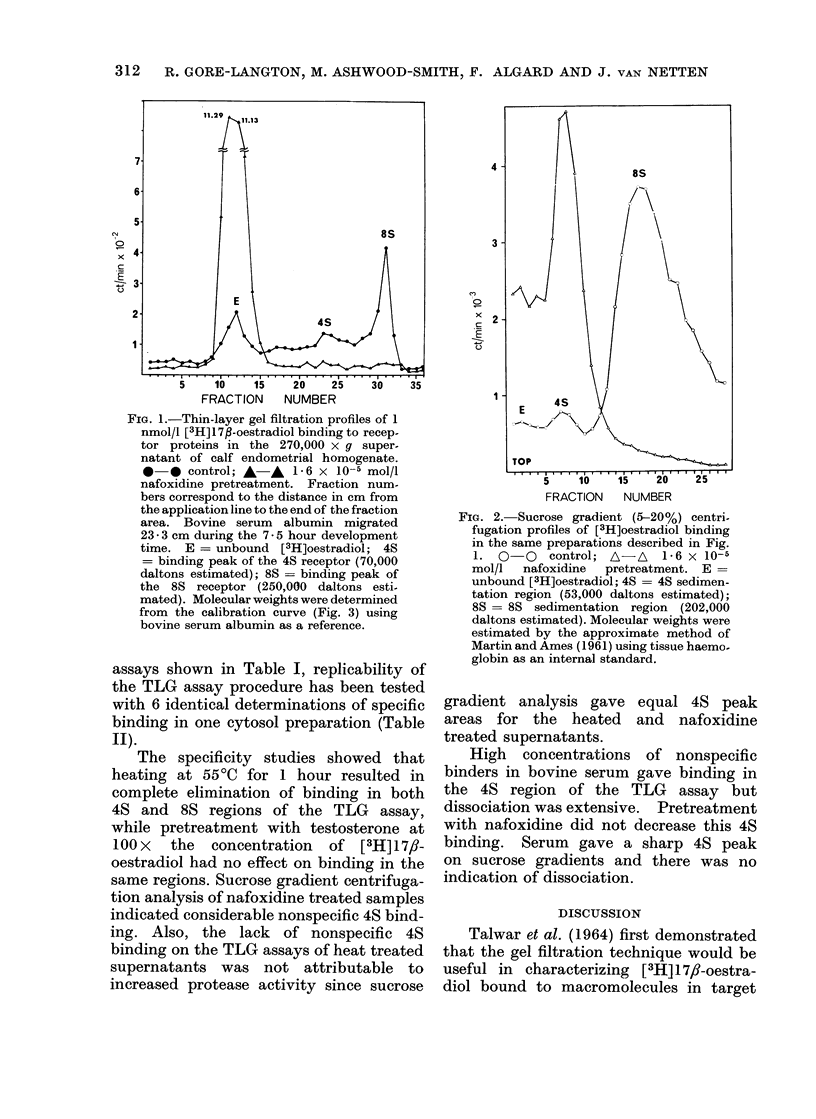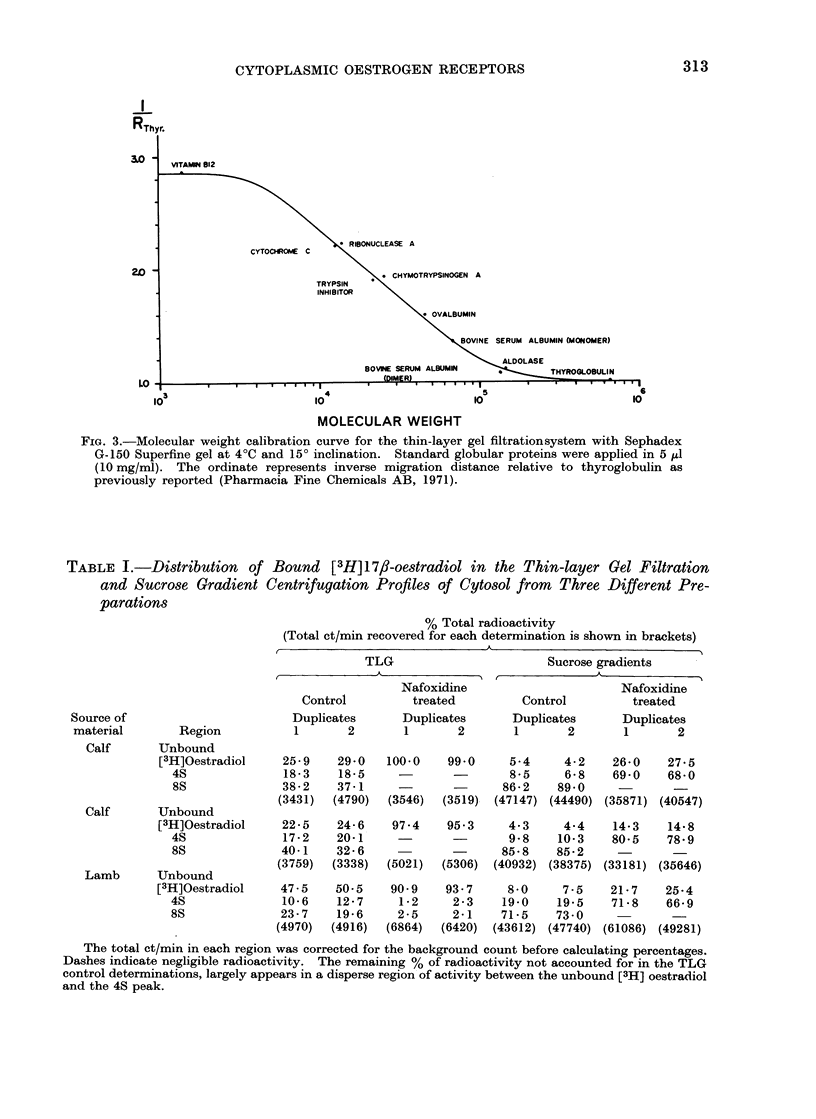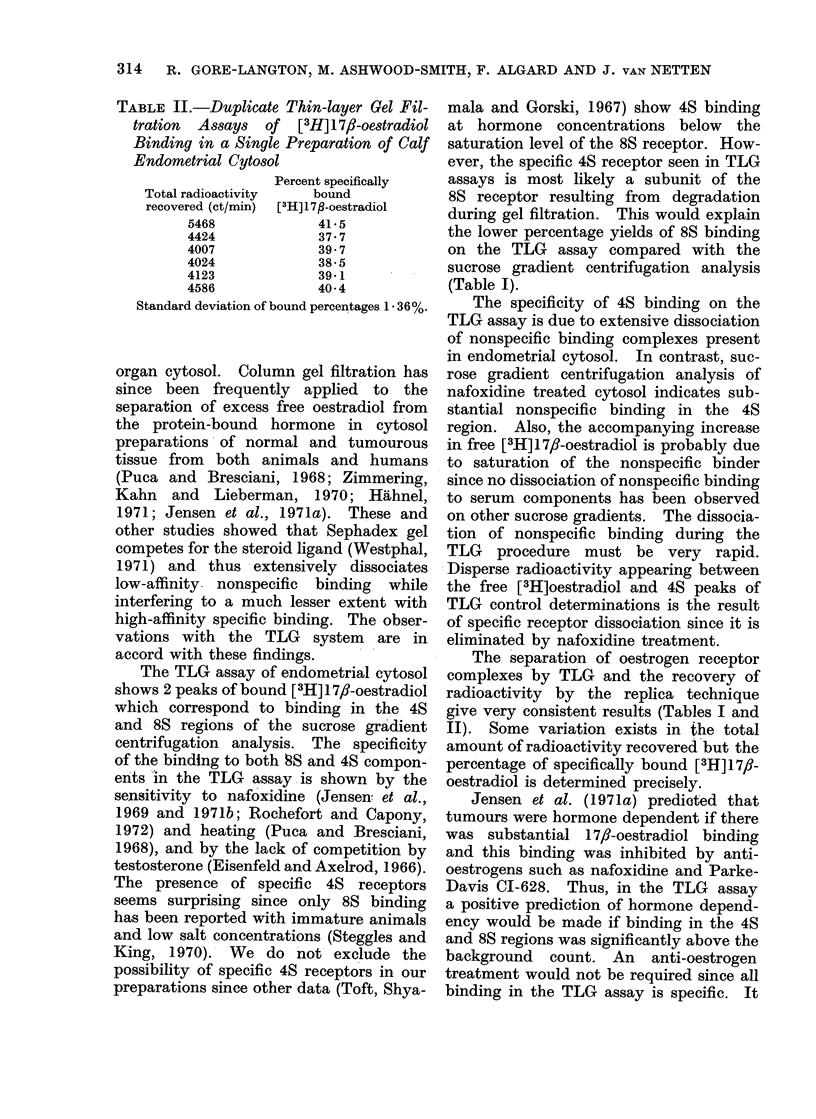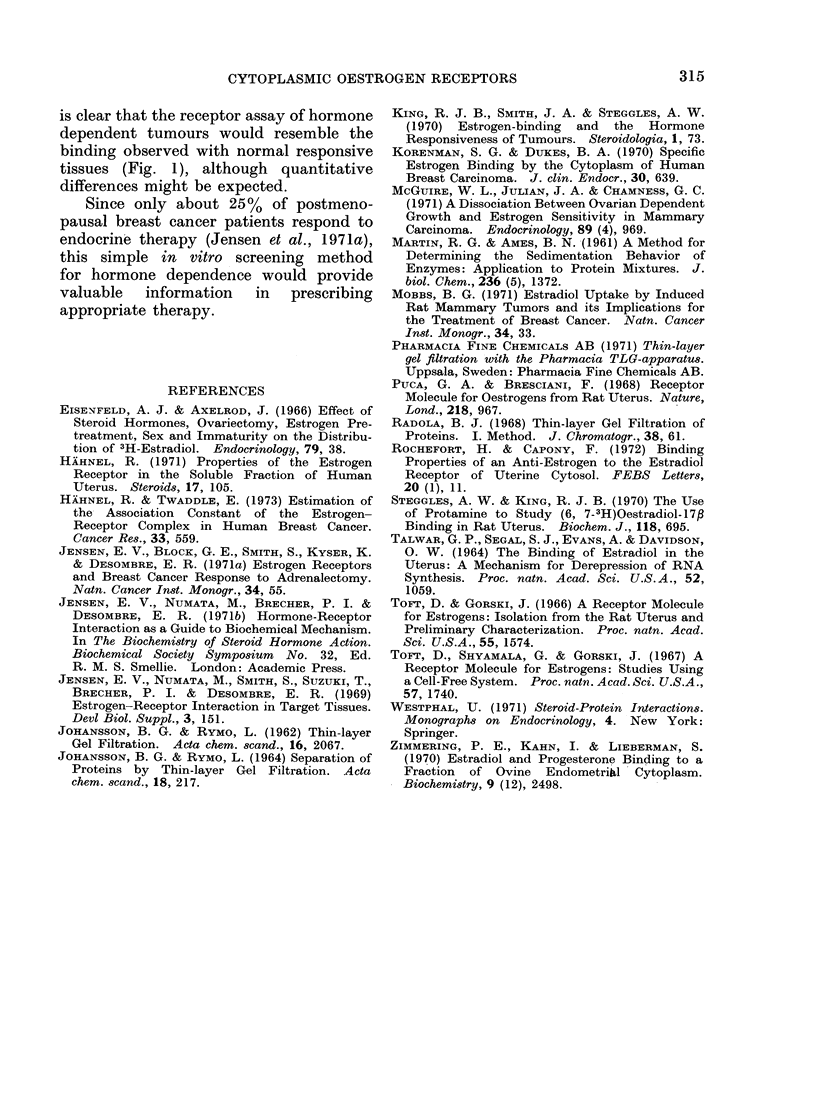# A Thin-layer Gel Filtration Assay of Cytoplasmic Oestrogen Receptors. A Possible Screening Method for Hormone Dependent Tumours

**DOI:** 10.1038/bjc.1973.153

**Published:** 1973-10

**Authors:** R. E. Gore-Langton, M. J. Ashwood-Smith, F. T. Algard, J. P. van Netten

## Abstract

A thin-layer gel filtration system for the assay of oestrogen receptors in target organ tissue samples as small as 50 mg is presented and compared with the sucrose gradient centrifugation method. Only specific high-affinity binding of [^3^H]17β-oestradiol to endometrial cytosol has been observed with the thin-layer gel filtration assay, despite the presence of relatively high levels of nonspecific binding components. The system could be adapted to the clinical determination of oestrogen receptor content in biopsy material from mammary and endometrial tumours and would be of value in predicting hormone dependency.


*Br. J. Cancer* (1973) **28**, 310

### A THIN-LAYER GEL FILTRATION ASSAY OF CYTOPLASMIC OESTROGEN RECEPTORS. A POSSIBLE SCREENING METHOD FOR HORMONE DEPENDENT TUMOURS

R. E. GORE-LANGTON, M. J. ASHWOOD-SMITH, F. T. ALGARD AND J. P. VAN NETTEN

*From the Department of Biological Sciences, University of Victoria, Victoria, British Columbia, Canada*

Received 12 March 1973. Accepted 25 June 1973

**Summary.—A thin-layer gel filtration system for the assay of oestrogen receptors in target organ tissue samples as small as 50 mg is presented and compared with the sucrose gradient centrifugation method. Only specific high-affinity binding of [$^3$H]17β-oestradiol to endometrial cytosol has been observed with the thin-layer gel filtration assay, despite the presence of relatively high levels of nonspecific binding components. The system could be adapted to the clinical determination of oestrogen receptor content in biopsy material from mammary and endometrial tumours and would be of value in predicting hormone dependency.**

A CLINICALLY useful correlation exists between the presence of high-affinity binding of [$^3$H]17*β*-oestradiol to oestrogen receptors in tumour tissue and the chances of response to endocrine therapy in breast cancer patients (Jensen *et al.*, 1971*a*). Also, the relationship between oestrogen receptor content and hormone dependency has been observed in certain other neoplastic target tissues (King, Smith and Steggles, 1970; McGuire, Julian and Chamness, 1971; Mobbs, 1971). Thus, a simple and reliable *in vitro* assay for oestrogen receptors would be valuable in predicting the effect of endocrine therapy *a priori*.

The separation of free from protein-bound [$^3$H]oestradiol by dextran coated charcoal adsorption procedures (Korenman and Dukes, 1970; Hähnel, 1971; Hähnel and Twaddle, 1973) appears to be more suitable for routine clinical application than other reported assays. Thin-layer gel filtration (TLG) is a reliable alternative to the dextran–charcoal procedures. Both methods have the advantages of simplicity, use of low-speed supernatants and feasibility of performing several simultaneous assays. In addition, TLG directly distinguishes between two high-affinity specific binding components and lower affinity nonspecific binding in endometrial cytosol incubated with $10^{-9}$ mol/l [$^3$H]17*β*-oestradiol. The dextran–charcoal procedures appear to dissociate most nonspecific binding under similar conditions, but generally specific and nonspecific binding must be distinguished by inference from a Scatchard plot. Further, the TLG assay has a distinct advantage in requiring only a 50 mg biopsy sample compared with about 1 g for the dextran–charcoal procedures of Hähnel and Twaddle (1973).

Sucrose density-gradient centrifugation provides good resolution of 4S and 8S binding components (Toft and Gorski, 1966; Steggles and King, 1970) and has therefore been employed for comparison with the TLG assay of the two corresponding oestrogen receptors.

### MATERIALS AND METHODS

Tissue preparation was according to the following procedure when supernatants were used for both TLG and sucrose gradient centrifugation assays. Calf or lamb uteri were transported on ice and processed at

0–2°C within 1 hour. About 3 g of endometrial tissue was finely minced in the presence of 0·2 ml of 40 mmol/l Tris HCl–1·5 mmol/l EDTA (Tris-EDTA) buffer, pH 7·4. A 30% (w/v) homogenate was prepared in Tris-EDTA buffer using a Potter–Elvejhem teflon-glass homogenizer. Supernatants obtained by centrifugation at 270,000 *g* for 1 hour were incubated in the absence (control) and presence of $1{\cdot}6 \times 10^{-5}$ mol/l nafoxidine (U11,100 courtesy of the Upjohn Co.). After 20 min [$^3$H]17$\beta$-oestradiol (2, 4, 6, 7 $^3$H, 106 Ci/mmol, New England Nuclear) was added ($10^{-9}$ mol/l) and incubation continued a further 30 min.

Supernatant preparation from 50 mg of tissue, for analysis by TLG only, consisted of a brief homogenization with 0·1 ml of Tris-EDTA buffer in a small glass homogenizer followed by direct centrifugation of the homogenization tube at 800 *g* for 15 min. The supernatant was transferred by micropipette to a glass tube and incubated with [$^3$H]17$\beta$-oestradiol as before.

Thin-layer gel filtration was performed using the Pharmacia TLG-apparatus (Pharmacia Fine Chemicals) with glass plates 20 × 40 cm. General considerations of the TLG method have been reported previously (Johansson and Rymo, 1962 and 1964; Radola, 1968; Pharmacia Fine Chemicals AB, 1971). Sephadex G-150 Superfine gel prepared in Tris-EDTA buffer was spread to a thickness of 0·6 mm. The gel layer was connected to the upper and lower eluant reservoirs by Whatman (3 MM) filter paper bridges and equilibration was performed at 22°C and 10° inclination. Supernatant samples of 20 $\mu$l were applied at 3 cm intervals along the origin of the precooled gel plate. TLG development was continued at 15° inclination and 0–4°C until a marker of bovine serum albumin (10 mg/ml) conjugated to amido black had migrated approximately 23 cm from the origin (about 7·5 hours). Radioactivity was recovered from the gel plate using a replica technique with No. 3 filter paper similar to that described by Radola (1968) for nonradioactive proteins. Filter paper bridges were removed before applying paper replicas to the gel surface for 15 min. The dried replica was cut into a 3·0 cm wide strip for each sample and 36 1·0 cm fractions were prepared. Paper fractions were placed in scintillation vials and radioactivity was determined in a Beckman Model LS133 liquid scintillation counter using 0·5% 2,5-diphenyloxazole (PPO) in toluene.

Supernatant samples of 0·3 ml on 5–20% sucrose gradients prepared in Tris-EDTA buffer were centrifuged in a Beckman SW56 rotor at either 40,000 rev/min for 17 hours or at 35,000 rev/min for 21 hours. Fractions were collected 5 drops per fraction from the top and radioactivity was determined as before.

Specificity studies were varied. Endometrial supernatants were treated with either heat at 55°C for 1 hour in the presence of $10^{-9}$ mol/l [$^3$H]17$\beta$-oestradiol or with $10^{-7}$ mol/l testosterone before incubation with $10^{-9}$ mol/l [$^3$H]17$\beta$-oestradiol, then assayed by TLG. Sucrose gradient centrifugation of control and nafoxidine treated supernatants was performed as a means of indicating the amount of nonspecific binding present. In addition, heat treated supernatants were centrifuged on sucrose gradients along with an unheated nafoxidine treated sample in order to check for degradation of the nonspecific binder due to increased protease activity during the heat treatment. In another experiment, a high concentration of nonspecific binders in bovine serum was incubated with [$^3$H]17$\beta$-oestradiol (with and without nafoxidine pretreatment) then assayed by TLG and sucrose gradient centrifugation.

### RESULTS

The distribution of bound [$^3$H]17$\beta$-oestradiol to the 270,000 *g* endometrial supernatant from 3 sources has been determined by TLG and sucrose gradient centrifugation analysis (Table I). [$^3$H]17$\beta$-oestradiol binding profiles for single duplicates of the first experiment in Table I are shown in Fig. 1 (TLG) and Fig. 2 (sucrose gradient centrifugation). The results of the 2 different assays were obtained using the same preparation of supernatant plus [$^3$H]17$\beta$-oestradiol and thus differences in binding are directly attributable to the assay systems. TLG analysis of low-speed supernatants prepared from 50 mg of tissue gave results equivalent to Fig. 1.

In addition to the duplicate TLG

FIG. 1.—Thin-layer gel filtration profiles of 1 nmol/l [$^3$H]17$\beta$-oestradiol binding to receptor proteins in the 270,000 × $g$ supernatant of calf endometrial homogenate. ●—● control; ▲—▲ 1·6 × $10^{-5}$ mol/l nafoxidine pretreatment. Fraction numbers correspond to the distance in cm from the application line to the end of the fraction area. Bovine serum albumin migrated 23·3 cm during the 7·5 hour development time. E = unbound [$^3$H]oestradiol; 4S = binding peak of the 4S receptor (70,000 daltons estimated); 8S = binding peak of the 8S receptor (250,000 daltons estimated). Molecular weights were determined from the calibration curve (Fig. 3) using bovine serum albumin as a reference.

FIG. 2.—Sucrose gradient (5–20%) centrifugation profiles of [$^3$H]oestradiol binding in the same preparations described in Fig. 1. ○—○ control; △—△ 1·6 × $10^{-5}$ mol/l nafoxidine pretreatment. E = unbound [$^3$H]oestradiol; 4S = 4S sedimentation region (53,000 daltons estimated); 8S = 8S sedimentation region (202,000 daltons estimated). Molecular weights were estimated by the approximate method of Martin and Ames (1961) using tissue haemoglobin as an internal standard.

assays shown in Table I, replicability of the TLG assay procedure has been tested with 6 identical determinations of specific binding in one cytosol preparation (Table II).

The specificity studies showed that heating at 55°C for 1 hour resulted in complete elimination of binding in both 4S and 8S regions of the TLG assay, while pretreatment with testosterone at 100× the concentration of [$^3$H]17$\beta$-oestradiol had no effect on binding in the same regions. Sucrose gradient centrifugation analysis of nafoxidine treated samples indicated considerable nonspecific 4S binding. Also, the lack of nonspecific 4S binding on the TLG assays of heat treated supernatants was not attributable to increased protease activity since sucrose gradient analysis gave equal 4S peak areas for the heated and nafoxidine treated supernatants.

High concentrations of nonspecific binders in bovine serum gave binding in the 4S region of the TLG assay but dissociation was extensive. Pretreatment with nafoxidine did not decrease this 4S binding. Serum gave a sharp 4S peak on sucrose gradients and there was no indication of dissociation.

### DISCUSSION

Talwar *et al.* (1964) first demonstrated that the gel filtration technique would be useful in characterizing [$^3$H]17$\beta$-oestradiol bound to macromolecules in target

FIG. 3.—Molecular weight calibration curve for the thin-layer gel filtrationsystem with Sephadex G-150 Superfine gel at 4°C and 15° inclination. Standard globular proteins were applied in 5 μl (10 mg/ml). The ordinate represents inverse migration distance relative to thyroglobulin as previously reported (Pharmacia Fine Chemicals AB, 1971).

TABLE I.—*Distribution of Bound* [$^3H$]17β-*oestradiol in the Thin-layer Gel Filtration and Sucrose Gradient Centrifugation Profiles of Cytosol from Three Different Preparations*

| | | % Total radioactivity (Total ct/min recovered for each determination is shown in brackets) | | | | | | | |
|---|---|---|---|---|---|---|---|---|---|
| | | TLG | | | | Sucrose gradients | | | |
| | | Control | | Nafoxidine treated | | Control | | Nafoxidine treated | |
| Source of material | Region | Duplicates 1 | 2 | Duplicates 1 | 2 | Duplicates 1 | 2 | Duplicates 1 | 2 |
| Calf | Unbound [$^3$H]Oestradiol | 25·9 | 29·0 | 100·0 | 99·0 | 5·4 | 4·2 | 26·0 | 27·5 |
| | 4S | 18·3 | 18·5 | — | — | 8·5 | 6·8 | 69·0 | 68·0 |
| | 8S | 38·2 | 37·1 | — | — | 86·2 | 89·0 | — | — |
| | | (3431) | (4790) | (3546) | (3519) | (47147) | (44490) | (35871) | (40547) |
| Calf | Unbound [$^3$H]Oestradiol | 22·5 | 24·6 | 97·4 | 95·3 | 4·3 | 4·4 | 14·3 | 14·8 |
| | 4S | 17·2 | 20·1 | — | — | 9·8 | 10·3 | 80·5 | 78·9 |
| | 8S | 40·1 | 32·6 | — | — | 85·8 | 85·2 | — | — |
| | | (3759) | (3338) | (5021) | (5306) | (40932) | (38375) | (33181) | (35646) |
| Lamb | Unbound [$^3$H]Oestradiol | 47·5 | 50·5 | 90·9 | 93·7 | 8·0 | 7·5 | 21·7 | 25·4 |
| | 4S | 10·6 | 12·7 | 1·2 | 2·3 | 19·0 | 19·5 | 71·8 | 66·9 |
| | 8S | 23·7 | 19·6 | 2·5 | 2·1 | 71·5 | 73·0 | — | — |
| | | (4970) | (4916) | (6864) | (6420) | (43612) | (47740) | (61086) | (49281) |

The total ct/min in each region was corrected for the background count before calculating percentages. Dashes indicate negligible radioactivity. The remaining % of radioactivity not accounted for in the TLG control determinations, largely appears in a disperse region of activity between the unbound [$^3$H] oestradiol and the 4S peak.

TABLE II.—*Duplicate Thin-layer Gel Filtration Assays of* [$^3$*H*]17*β-oestradiol Binding in a Single Preparation of Calf Endometrial Cytosol*

| Total radioactivity recovered (ct/min) | Percent specifically bound [$^3$H]17β-oestradiol |
|---|---|
| 5468 | 41·5 |
| 4424 | 37·7 |
| 4007 | 39·7 |
| 4024 | 38·5 |
| 4123 | 39·1 |
| 4586 | 40·4 |

Standard deviation of bound percentages 1·36%.

organ cytosol. Column gel filtration has since been frequently applied to the separation of excess free oestradiol from the protein-bound hormone in cytosol preparations of normal and tumourous tissue from both animals and humans (Puca and Bresciani, 1968; Zimmering, Kahn and Lieberman, 1970; Hähnel, 1971; Jensen *et al.*, 1971*a*). These and other studies showed that Sephadex gel competes for the steroid ligand (Westphal, 1971) and thus extensively dissociates low-affinity nonspecific binding while interfering to a much lesser extent with high-affinity specific binding. The observations with the TLG system are in accord with these findings.

The TLG assay of endometrial cytosol shows 2 peaks of bound [$^3$H]17β-oestradiol which correspond to binding in the 4S and 8S regions of the sucrose gradient centrifugation analysis. The specificity of the binding to both 8S and 4S components in the TLG assay is shown by the sensitivity to nafoxidine (Jensen *et al.*, 1969 and 1971*b*; Rochefort and Capony, 1972) and heating (Puca and Bresciani, 1968), and by the lack of competition by testosterone (Eisenfeld and Axelrod, 1966). The presence of specific 4S receptors seems surprising since only 8S binding has been reported with immature animals and low salt concentrations (Steggles and King, 1970). We do not exclude the possibility of specific 4S receptors in our preparations since other data (Toft, Shyamala and Gorski, 1967) show 4S binding at hormone concentrations below the saturation level of the 8S receptor. However, the specific 4S receptor seen in TLG assays is most likely a subunit of the 8S receptor resulting from degradation during gel filtration. This would explain the lower percentage yields of 8S binding on the TLG assay compared with the sucrose gradient centrifugation analysis (Table I).

The specificity of 4S binding on the TLG assay is due to extensive dissociation of nonspecific binding complexes present in endometrial cytosol. In contrast, sucrose gradient centrifugation analysis of nafoxidine treated cytosol indicates substantial nonspecific binding in the 4S region. Also, the accompanying increase in free [$^3$H]17β-oestradiol is probably due to saturation of the nonspecific binder since no dissociation of nonspecific binding to serum components has been observed on other sucrose gradients. The dissociation of nonspecific binding during the TLG procedure must be very rapid. Disperse radioactivity appearing between the free [$^3$H]oestradiol and 4S peaks of TLG control determinations is the result of specific receptor dissociation since it is eliminated by nafoxidine treatment.

The separation of oestrogen receptor complexes by TLG and the recovery of radioactivity by the replica technique give very consistent results (Tables I and II). Some variation exists in the total amount of radioactivity recovered but the percentage of specifically bound [$^3$H]17β-oestradiol is determined precisely.

Jensen *et al.* (1971*a*) predicted that tumours were hormone dependent if there was substantial 17β-oestradiol binding and this binding was inhibited by anti-oestrogens such as nafoxidine and Parke-Davis CI-628. Thus, in the TLG assay a positive prediction of hormone dependency would be made if binding in the 4S and 8S regions was significantly above the background count. An anti-oestrogen treatment would not be required since all binding in the TLG assay is specific. It is clear that the receptor assay of hormone dependent tumours would resemble the binding observed with normal responsive tissues (Fig. 1), although quantitative differences might be expected.

Since only about 25% of postmenopausal breast cancer patients respond to endocrine therapy (Jensen *et al.*, 1971*a*), this simple *in vitro* screening method for hormone dependence would provide valuable information in prescribing appropriate therapy.

### REFERENCES

EISENFELD, A. J. & AXELROD, J. (1966) Effect of Steroid Hormones, Ovariectomy, Estrogen Pretreatment, Sex and Immaturity on the Distribution of $^3$H-Estradiol. *Endocrinology*, **79**, 38.

HÄHNEL, R. (1971) Properties of the Estrogen Receptor in the Soluble Fraction of Human Uterus. *Steroids*, **17**, 105.

HÄHNEL, R. & TWADDLE, E. (1973) Estimation of the Association Constant of the Estrogen–Receptor Complex in Human Breast Cancer. *Cancer Res.*, **33**, 559.

JENSEN, E. V., BLOCK, G. E., SMITH, S., KYSER, K. & DESOMBRE, E. R. (1971*a*) Estrogen Receptors and Breast Cancer Response to Adrenalectomy. *Natn. Cancer Inst. Monogr.*, **34**, 55.

JENSEN, E. V., NUMATA, M., BRECHER, P. I. & DESOMBRE, E. R. (1971*b*) Hormone-Receptor Interaction as a Guide to Biochemical Mechanism. In *The Biochemistry of Steroid Hormone Action. Biochemical Society Symposium No.* 32, Ed. R. M. S. Smellie. London: Academic Press.

JENSEN, E. V., NUMATA, M., SMITH, S., SUZUKI, T., BRECHER, P. I. & DESOMBRE, E. R. (1969) Estrogen–Receptor Interaction in Target Tissues. *Devl Biol. Suppl.*, **3**, 151.

JOHANSSON, B. G. & RYMO, L. (1962) Thin-layer Gel Filtration. *Acta chem. scand.*, **16**, 2067.

JOHANSSON, B. G. & RYMO, L. (1964) Separation of Proteins by Thin-layer Gel Filtration. *Acta chem. scand.*, **18**, 217.

KING, R. J. B., SMITH, J. A. & STEGGLES, A. W. (1970) Estrogen-binding and the Hormone Responsiveness of Tumours. *Steroidologia*, **1**, 73.

KORENMAN, S. G. & DUKES, B. A. (1970) Specific Estrogen Binding by the Cytoplasm of Human Breast Carcinoma. *J. clin. Endocr.*, **30**, 639.

MCGUIRE, W. L., JULIAN, J. A. & CHAMNESS, G. C. (1971) A Dissociation Between Ovarian Dependent Growth and Estrogen Sensitivity in Mammary Carcinoma. *Endocrinology*, **89** (4), 969.

MARTIN, R. G. & AMES, B. N. (1961) A Method for Determining the Sedimentation Behavior of Enzymes: Application to Protein Mixtures. *J. biol. Chem.*, **236** (5), 1372.

MOBBS, B. G. (1971) Estradiol Uptake by Induced Rat Mammary Tumors and its Implications for the Treatment of Breast Cancer. *Natn. Cancer Inst. Monogr.*, **34**, 33.

PHARMACIA FINE CHEMICALS AB (1971) *Thin-layer gel filtration with the Pharmacia TLG-apparatus*. Uppsala, Sweden: Pharmacia Fine Chemicals AB.

PUCA, G. A. & BRESCIANI, F. (1968) Receptor Molecule for Oestrogens from Rat Uterus. *Nature, Lond.*, **218**, 967.

RADOLA, B. J. (1968) Thin-layer Gel Filtration of Proteins. I. Method. *J. Chromatogr.*, **38**, 61.

ROCHEFORT, H. & CAPONY, F. (1972) Binding Properties of an Anti-Estrogen to the Estradiol Receptor of Uterine Cytosol. *FEBS Letters*, **20** (1), 11.

STEGGLES, A. W. & KING, R. J. B. (1970) The Use of Protamine to Study (6, 7-$^3$H)Oestradiol-17$\beta$ Binding in Rat Uterus. *Biochem. J.*, **118**, 695.

TALWAR, G. P., SEGAL, S. J., EVANS, A. & DAVIDSON, O. W. (1964) The Binding of Estradiol in the Uterus: A Mechanism for Derepression of RNA Synthesis. *Proc. natn. Acad. Sci. U.S.A.*, **52**, 1059.

TOFT, D. & GORSKI, J. (1966) A Receptor Molecule for Estrogens: Isolation from the Rat Uterus and Preliminary Characterization. *Proc. natn. Acad. Sci. U.S.A.*, **55**, 1574.

TOFT, D., SHYAMALA, G. & GORSKI, J. (1967) A Receptor Molecule for Estrogens: Studies Using a Cell-Free System. *Proc. natn. Acad. Sci. U.S.A.*, **57**, 1740.

WESTPHAL, U. (1971) *Steroid-Protein Interactions. Monographs on Endocrinology*, **4**. New York: Springer.

ZIMMERING, P. E., KAHN, I. & LIEBERMAN, S. (1970) Estradiol and Progesterone Binding to a Fraction of Ovine Endometrial Cytoplasm. *Biochemistry*, **9** (12), 2498.